# A Study on the Interfacial Compatibility, Microstructure and Physico-Chemical Properties of Polyimide/Organically Modified Silica Nanocomposite Membrane

**DOI:** 10.3390/polym13081328

**Published:** 2021-04-18

**Authors:** Md A. Wahab, Mohammad R. Karim, Muhammad O. Aijaz, Bidita Salahuddin, Shazed Aziz, Abu A. I. Sina

**Affiliations:** 1 Institute for Advanced Study, Chengdu University, Chengdu 610106, China; 2 Institute of Materials Research and Engineering (IMRE) of The Agency for Science, Technology, and Research (A*STAR), 3 Research Link, Singapore 117602, Singapore; 3 Center of Excellence for Research in Engineering Materials (CEREM), Deanship of Scientific Research (DSR), King Saud University, Riyadh 11421, Saudi Arabia; mkarim@ksu.edu.sa (M.R.K.); maijaz@ksu.edu.sa (M.O.A.); 4 K. A. CARE Energy Research and Innovation Center, Riyadh 11451, Saudi Arabia; 5 ARC Centre of Excellence for Electromaterials Science and Intelligent Polymer Research Institute, Innovation Campus, University of Wollongong, Squires Way, North Wollongong, NSW 2522, Australia; bbs622@uowmail.edu.au; 6 School of Chemical Engineering, The University of Queensland, QLD 4072, Australia; 7 Centre for Personalized Nanomedicine, Australian Institute for Bioengineering and Nanotechnology (AIBN), The University of Queensland, QLD 4072, Australia

**Keywords:** composites, polyimide, silica, sol-gel method, 3-glycidoxypropyltrimethoxysilane, ^29^Si NMR, interfacial interactions, compatibility

## Abstract

Polyimide-silica (PI-Silica) composites are of tremendous research interest as high-performance materials because of their excellent thermal and mechanical properties and chemical resistance to organic solvents. Particularly, the sol-gel method of fabricating such composites is popular for manipulating their properties. In this work, PI-silica composite films are synthesized by the sol-gel method and thermal imidization from the solution mixtures of hydrolyzed tetraethoxysilane (TEOS) (or glycidoxypropyltrimethoxysilane (GPMS)) modified silica and an aromatic polyamic acid (PAA) based on 3,3′,4,4′-biphenyl tetracarboxylic dianhydride (BPDA)–p-phenylenediamine (PDA). The phase morphology of composites is found to be controlled by the substitution of TEOS with GPMS. Solid-state NMR spectroscopy is used to confirm the structural components of silica and GPMS-modified silica, whereas FT-IR results confirm the complete imidization of polyimide and composite film and suggest successful incorporation of Si–O–Si bonds into polyimide. The thermal, optical transmittance, and dielectric constant characterizations of pure polyimide and composite films are also carried out. Thermal stability of pure polyimide is found to be increased significantly by the addition of silica, whereas the partial substitution of TEOS with GPMS decreases the thermal stability of the composite, due to the presence of the alkyl organic segment of GPMS. The optical transmittance and dielectric constant of the composite films are controlled by manipulating the GPMS content.

## 1. Introduction

Polyimide (PI) materials exhibit outstanding physico-chemical, electrical, and mechanical properties that allow them to be used in several advanced applications such as microelectronic, packaging, and separation [1,2,3,4,5,6,7,8]. Among polymers, particularly, PIs are a very important class of polymers because the organic segments of these polymers can easily be changed in length, rigidity, geometry of substitution, and functionality so that the overall properties such as transparency, thermal and mechanical, compatibility, processability, dielectric constant, and chemical resistance can easily be tuned [9,10,11]. It is found that the addition of reinforcing filler into the PI matrix has also shown to provide such desirable properties. Therefore, these composites with interesting synergistic properties have received much attention from both academics and industries. These composites have consisted of the organic polymer matrix and either organic or inorganic fillers [12,13,14,15]. For instance, polyimide-silica (PI-silica) composites are widely used as structural materials, in which, silica is used as an inorganic filler. Based on the studies, the type of silica fillers (either organosilica/inorganic) can control the compatibility and thermo-mechanical properties of the final PI matrix. Therefore, the use of those composites could easily be extended to the above-mentioned applications as well as other suitable applications including automotive, aerospace, and high-temperature manufacturing facilities. For making composites, the incorporation of various amounts of inorganic silica into the PI polymer has been a well-practiced strategy that enhances the thermomechanical properties of the polymer [16,17,18,19]. Most of the previous studies have used the sol-gel reaction to incorporate inorganic silica as filler into polymer because of its relatively low processing temperatures and the wide range of metal-organic sources that can be introduced. However, the control of the phase separation between organic and inorganic phases is still the primary problem for the formation of PI-silica nanocomposites, which significantly affects the final morphology of target composites [19,20,21]. Most approaches have used various silane coupling agents for improving the phase compatibility between trialkoxysilane-based organosilica and polymer phase. The final morphology of composites is largely affected by the nature of organic functional groups in the final composite phases. The resulting composite with large phase separation is found to deteriorate the thermomechanical properties instead of enhancement [22,23,24]. To address this problem, Akter et al. [25,26] have reported the polyimide-silica composites, in which the newly synthesized oligomeric species 2,6-bis(3-(triethoxysilyl)propyl)pyrrolo[3,4-f]isoindole-1,3,5,7(2H,6H)-tetraone (APA) was used as a coupling agent to improve compatibility between organic and inorganic phases. The APA-modified the silica particles (TEOS) were able to create bonds between imide linkages and hydroxyl groups that have played a role in improving the morphology and thermo-mechanical properties because of interfacial interactions that occurred through H-bonding and also like-like chemical interactions.

It is well-known that hydroxyl groups of silanol (Si–OH) undergo reactions with acid groups of polyamic acid (PAA) [16,17,18,19,20,21,22,23,24,27]. So far, most of the interactions have been interpreted from the final imidized composites only using FT-IR and improved thermomechanical properties. However, the interpretation of the introduced silica materials before adding them into PAA has hardly been considered. Thus, it is important to explain why the condensation sites such as Q^n^ and T^n^ sites of silica and organosilica, respectively, are vital for controlling the phase separation for the formation of composite phases. 

Herein, we report the structural characterization and influence of the substitution of TEOS with GPMS to effectively control the phase separation of the final composites and the following outcomes could set (i) the synthesis and the ^29^Si CP MAS NMR characterization of inorganic silica filler (Q^n^ site) and organosilica-modified silica (T^n^ sites). Then, 3-glycidoxypropyl trimethoxysilane (GPMS) which contains propyl organic segment and hydrophilic silanols at the end along with tetraethoxysilane (TEOS) was used as filler; (ii) discuss the phase compatibility, optical transmittance, thermal properties, FT-IR, and dielectric constant of composite films.

## 2. Materials and Methods

### 2.1. Materials

Tetraethoxysilane (TEOS), 3-glycidoxypropyltrimethoxysilane (GPMS), 3,3′,4,4′-Biphenyltetracarboxylic dianhydride (BPDA), *p*-phenylenediamine (PDA) were purchased from Sigma Aldrich and used as received. Deionized water, technical grade ethanol, dilute HCl, and dimethylacetamide (DMAc) were also used in this study. 

### 2.2. Synthesis of BPDA-PDA PAA, PI-Silica, and PI-Organically Modified Silica Composite Films

**Figure 1:**
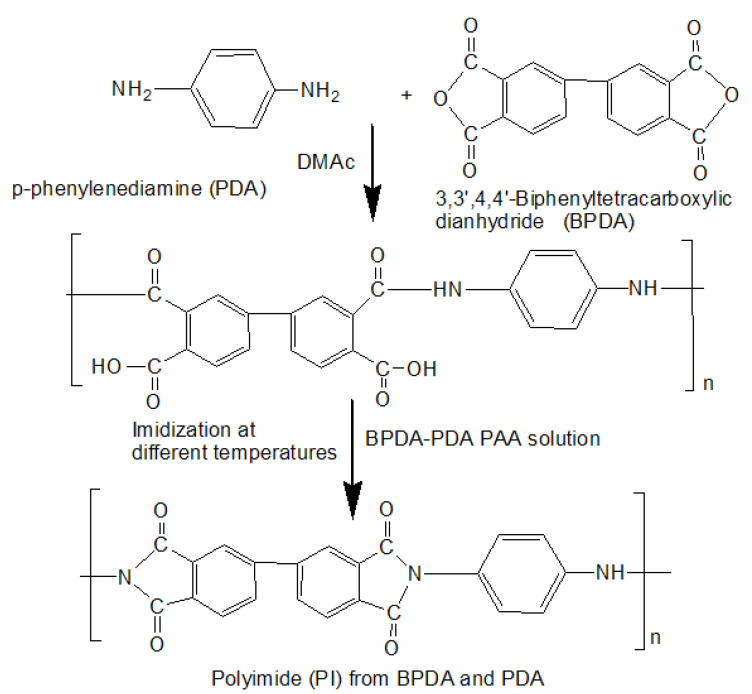
Synthesis of BPDA-PDA PAA solution and fabrication of PI film.

The 15-wt% of the BPDA-PDA PAA solution in DMAc was synthesized as shown in Figure 1 using the equimolar ratio of BPDA and PDA under nitrogen atmosphere. The solution was stirred for 24 h under nitrogen flow at room temperature to make a homogeneous solution. Then, the PAA solution was cast onto a glass plate by the applicator. Then, thermal imidization was also carried out at various temperatures (60 °C for 2 h, 80 °C for 2 h, 200 °C for 1 h, and 300 °C for 1.5 h) in a vacuum oven with nitrogen flow. The used heating rate was 2 °C/min. The chemical structures of BPDA and PDA and their conversion from monomers to PAA and BPDA–PDA PI are shown in Figure 1.

**Figure 2:**
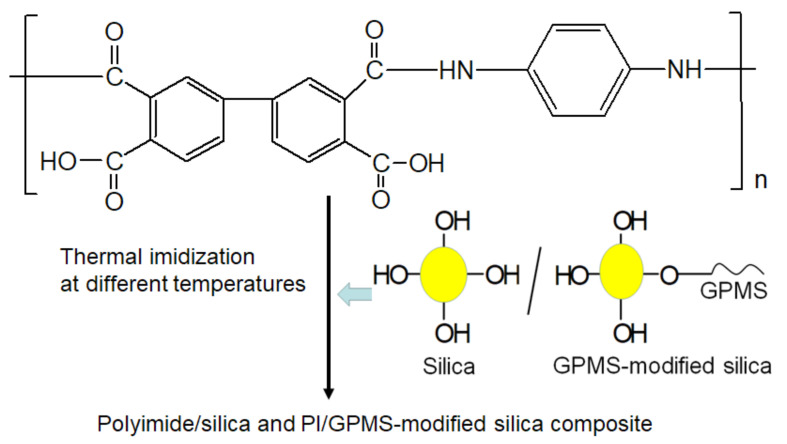
Synthesis of silica/GPMS-modified containing BPDA-PDA PAA composite solution and its conversion into polyimide/silica composite film and PI/GPMS-modified silica composite film.

**Figure 3:**
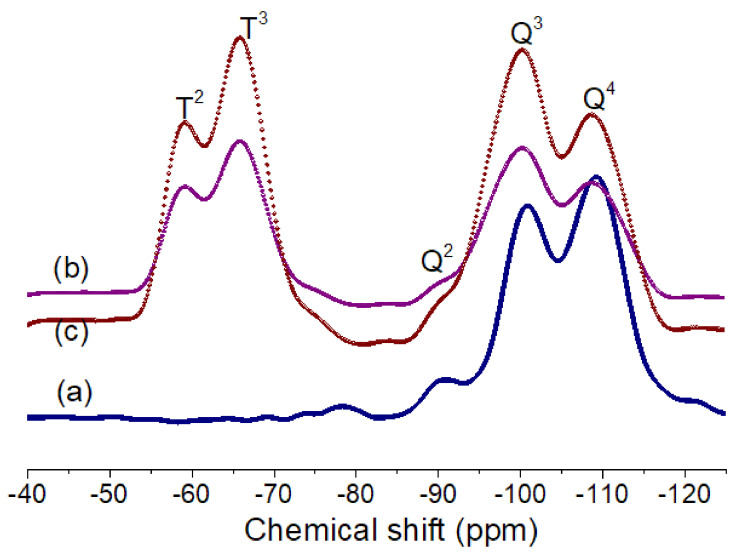
Solid-state ^29^Si MAS NMR spectra of silica and GPMS-modified silica samples: (**a**) TSi, (**b**) TSi-GPMS25, and (**c**) TSi-GPMS50.

**Figure 4:**
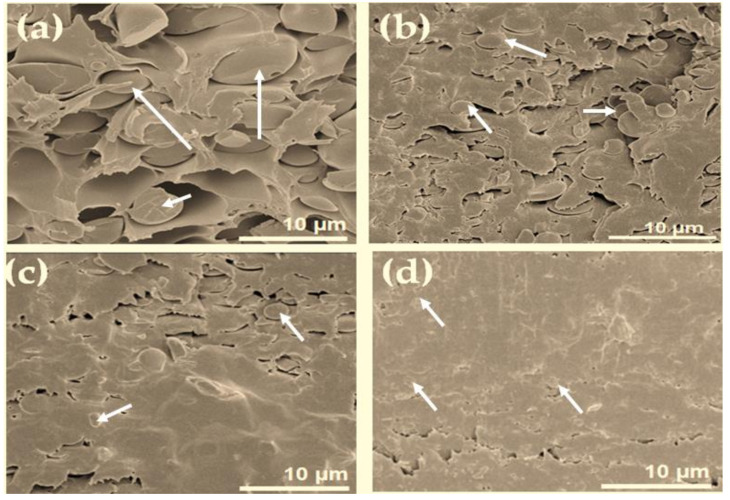
Cross-sectional fracture surface images of different composite samples: (**a**) PI-TSi, (**b**) PI-TSi-GPMS25, (**c**) PI-TSi-GPMS50, and (**d**) PI-TSi-GPMS75. Arrows show silica/GPMS-modified silica where the continuous matrix phase is polymer.

**Figure 5:**
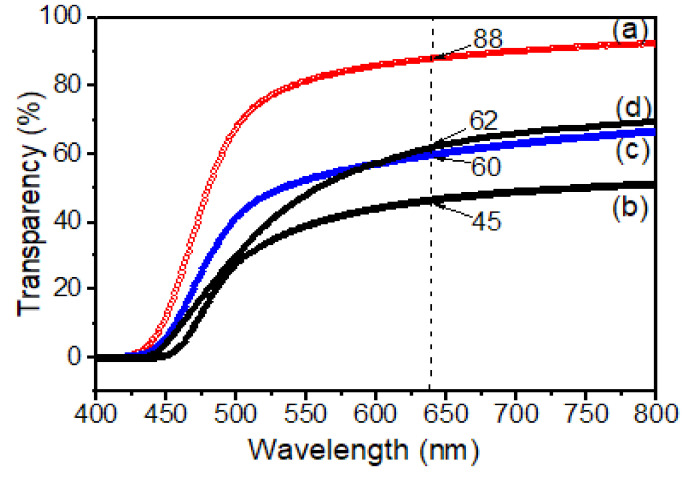
UV spectra of various samples: (**a**) pure PI, (**b**) PI-TSi; (**c**) PI-TSi-GPMS50; and (**d**) PI-TSi-GPMS75. The values of all films at 638 nm are inserted in Figure 5.

**Figure 6:**
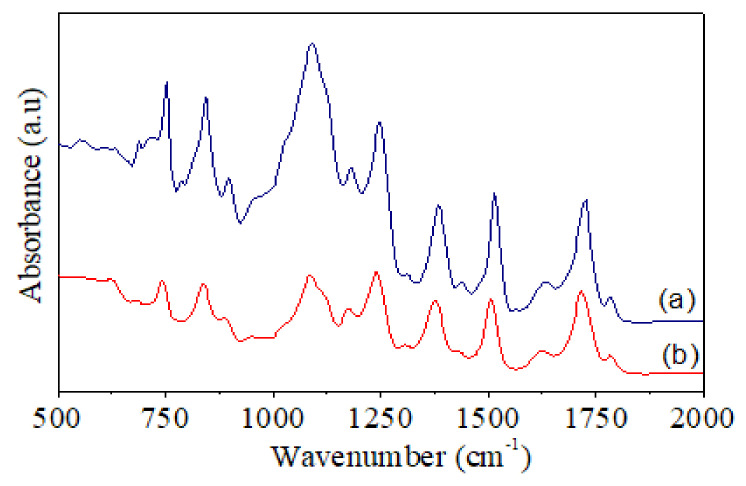
FT-IR spectra of (**a**) pure PI and (**b**) PI-TSi-GPMS50.

**Figure 7:**
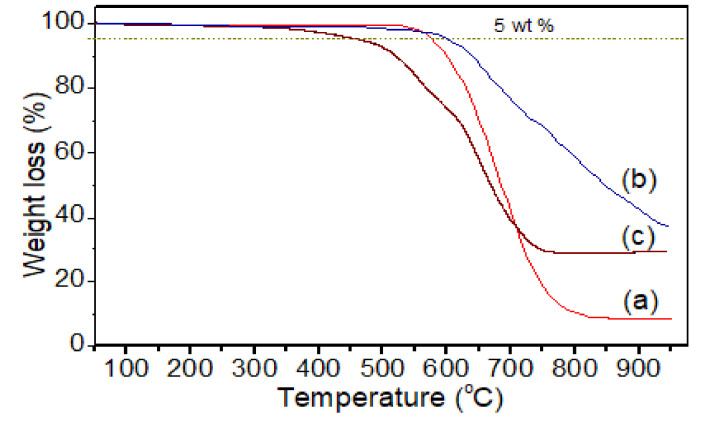
Thermal analysis of (**a**) pure PI, (**b**) PI-TSi, and (**c**) PI-TSi-GPMS50 composites.

**Figure 8:**
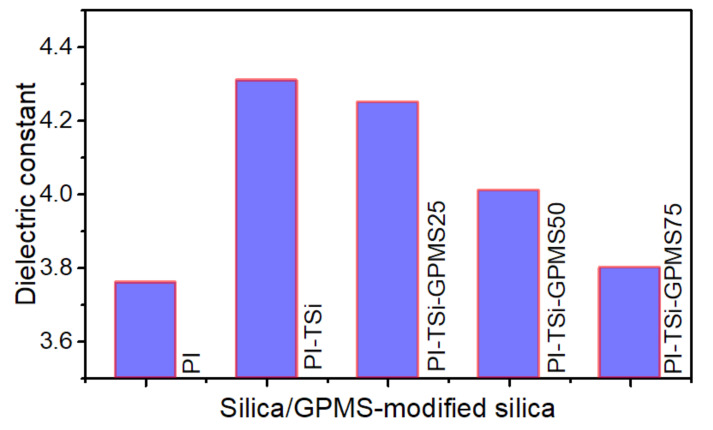
Effect of silica and GPMS-modified silica on the dielectric constant of polyimide.

For preparing PI/silica and PI/GPMS-modified silica composites, the 30 wt% of pre-prepared silica sol (TSi) was added drop by drop into PAA solution as shown in Figure 2 to avoid local inhomogeneities under nitrogen flow. Then composite PI-TSi/PI-TSi-GPMS containing BPDA-PDA PAA solution films was cast onto the glass plate and the films were imidized under the same heating protocol. Finally, after curing at different temperatures, free-standing composite films were successfully obtained. Sample codes, physical appearance, optical transmittance, and 5 wt% (*T*_d5_) of thermal decomposition of the various samples are shown in Table 1. The film thickness of all the films was about 200 µm. The spectroscopy, optical transmittance, microstructure, thermal and physico-chemical properties of the final composites are systematically discussed in Figure 3, Figure 4, Figure 5, Figure 6, Figure 7 and Figure 8.

### 2.3. Preparation of Pure Silica (TSi) and GPMS-Modified Silica (TSi-GPMS)

The silica sample was prepared by the drop-wise addition of TEOS to ethanol (1.3 mL) in the presence of 0.07M HCl (0.3 mL) and the stoichiometric amount of water against the amount of TEOS for making TSi and TSi-GPMS, whereas the amount of ethanol and HCl is kept constant for the all the samples. At first, ultrasonication was carried out to make the homogeneous solution of silica, and then stirring was done. Solution vials were stirred in a closed vial at room temperature before adding them into the BPDA-PDA PAA solution. In this study, we prepared silica and GPMS-modified silica by replacing the partial amount of TEOS with organosilica GPMS as shown in Figure 2. 

### 2.4. Characterization and Properties Evaluation

The solid-state NMR (Nuclear Magnetic Resonance) spectra were recorded on the same NMR instrument equipped with solid-state probe using the following experimental conditions: ^13^C CP/MAS NMR, 6 usec prescan delay time, 2000 usec contact pulse, 5 kHz MAS rate, and 2000 scans; ^29^Si CP/MAS NMR, 30.0 usec delay time, 1200 usec contact pulse, 5 kHz MAS rate, and 2500 scans. Then, a field-emission scanning electron microscope (FE-SEM) (JEOL) was used for observing the fractured cross-section of composite films. The fractured surfaces of the samples were obtained by dipping them into liquid nitrogen for 10 min and then broken into two pieces by radial impact force. The exposed fracture surfaces were gold-coated for 20 sec prior to SEM observation. UV spectra for the reported samples were recorded using a UV-Visible photo-spectrometer (UV-3101PC). Fourier Transform Infrared spectroscopy (FT-IR) spectra data were collected using a Per-kin-Elmer spectrometer using ATR (Attenuated Total Reflection) mode. Thermogravimetric analysis was carried using under nitrogen atmosphere on TA instrument (TGA Q500) by maintaining a heating rate of 15 °C min^−1^ from 50 to 950 °C. The dielectric constants (DC) of the films were recorded on a TA instrument (DEA 2970) analyzer with a heating rate of 5 °C/min.

## 3. Results and Discussion

### 3.1. Structural Characterization by NMR Spectroscopy

In this study, we prepared a few sets of samples such as pure silica (TSi), and GPMS-modified silica samples (TSi-GPMS25, TSi-GPMS50, and TSi-GPMS75) for investigating the compatibility between organic and inorganic phases (TSi-GPMS). The content of GPMS in the TEOS mixture was also varied for comparison. Figure 3 shows the ^29^Si NMR spectra of three samples, in which it is seen that silicon atoms are capable of forming a silica network with various Si–O bonds. Therefore, the Si-O bonds in the silica network are usually defined as Q^n^ and T^n^ sites, where the presence of Q^n^ type is due to the formation of pure silica network and T^n^-type is formed from the organosilica type material such as GPMS. It was previously demonstrated that the silica network could be assigned to Si(OSi)_4_ (Q^4^), Si(OSi)_3_OH (Q^3^), and Si(OSi)_2_(OH)_2_ Q^2^) [28,29,30,31,32,33]. The silica network with hydroxyl groups (-OH) could be further functionalized through available -OH groups. This study shows a few resonances in Figure 3a, which could be assigned as the ~109.01 ppm, for Q^4^ (Si(OSi)_4_), ~100.1 ppm for Q^3^ (Si(OSi)_3_OH), and ~90.13 ppm for Q^2^ Si(OSi)_2_OH), agreement with the previously reported values for silica structure [28,29,30,31,32,33]. For GPMS-modified silica (TSi-GPMS25), Figure 3b shows two peaks from the GPMS organosilica structures at about −65.94 ppm, and −59.13 ppm, which correspond to the T^3^ and T^2^, respectively, along with Q^n^ sites, suggesting that the co-condensation reaction of silica structure with GPMS has successfully taken place under the employed conditions and these T^n^ sites are consistent with previously published reports on silica and organosilane modified silica structures [28,29,30,31,32,33]. For the TSi-GPMS sample, it could be suggested that the GPMS-modified silica framework is formed through the formation of the Si-O-Si bond using the surface available hydroxyl groups of TEOS and GPMS as shown in Figure 2 [28,29,30,31,32,33]. 

### 3.2. Influence of GPMS on the Morphology of the Composite

The fracture surface SEM images in Figure 4 display that the addition of silica (TSi) and GPMS-modified silica structures (PI-TSi-GPMS25, PI-TSi-GPMS50, PI-TSi-GPMS75) into the polymer solution has affected the final phase morphology of composites prepared. The SEM image in Figure 4a is more easily distinguishable than that of Figure 4b–d in terms of particle size, distribution, and phase compatibility. In Figure 4a, the fairly large spherical particles are dispersed in the polymer phase, showing the poor interfacial compatibility between inorganic silica particles and the polymer phase. The final composite in Figure 4a also suggests the almost absence of strong hydrogen bonding sites in the system, consistent with previously reported silica-incorporated PI composites [6,12,20,21,34,35,36,37,38,39,40,41,42,43,44]. In contrast, GPMS-modified silica-based PI composites have shown improved morphology as well as compatibility between two phases. The images in Figure 4b,c are also displaying interconnected and well-dispersed spherical particles with reduced sizes in the continuous organic phase. This high level of phase compatibility is obtained when composites are prepared using the GPMS-modified silica solution. Among the cross-sectional SEM images, the phase compatibility in Figure 4c is better than that of the images in Figure 4b,4c because it has more GPMS (PI-TSi-GPMS75). Figure 4d also shows a very well-compatibilized composite with smaller particle sizes. 

The difference in phase compatibility could also be described due to the presence of more functional hydroxyl sites and also the organic segment of GPMS in the GPMS-modified silica sample. These images could also be supported by the ^29^Si NMR data in Figure 3. Figure 3a for the pure silica sample is dominated by the Q^4^ site which also indicates the presence of very less number of available hydroxyl groups that would undergo interaction with –COOH groups of PAA, whereas a higher number of hydroxyl groups in Figure 3b,c are available which can lead to the improved compatibilized composites with reduced particle sizes due to the strong hydrogen bonding interactions between –COOH groups of PAA and –OH groups of the GPMS-modified sample [27]. Also, the organic segment in GPMS can play a significant role in enhancing compatibility. Therefore, it is suggested that synergistic effects due to the presence of available hydroxyl groups and the organic segment of the GPMS-modified silica sample have preluded the shaping of the phase morphology of the final composites. For example, the composite phase morphology in Figure 4d for sample PI-TSi-GPMS75 shows highly intermingled compatibilized phases with a reduced particle size compared to that of other samples (PI-TSi-GPMS25 and PI-TSi-GPMSS50). A high level of phase compatibility was achieved when 75% (TSi-GPMS75 sample) of TEOS was replaced with GPTMS. These SEM images in Figure 4 verified that the extent of interfacial compatibility of the final composites depends on the amount of GPMS in the TEOS solution. It could also be suggested that the formation of sufficient quantities of functional groups (either TEOS or GPMS or TEOS-GPMS) including the hydroxyl group in the organic segment can induce the system to reach the required level of compatibilization [34,35,36,37,38,39,42,43,44]. 

### 3.3. Optical Transmittance

Figure 5 shows the optical transmittance (T%) of pure PI and composites at 638 nm. The pure PI was highly transparent which was supported by the value of optical transmittance (T%) (89%) at 638 nm. In contrast, for the composite film, the opaque PI-TSi composite film showed only 45% transmittance, indicating higher light scattering due to the increased particle sizes of silica as shown in Figure 4b. The physical observations of the films are also reported in Table 1. The optical transmittance (T%) in Table 1 and Figure 5 was found to be increased for PI-TSi-GPMS50, and PI-TSi-GPMS75 composites indicated the effects of increasing the amount of GPMS in the TEOS mixture that allow favorably good dispersion of silica particles in the polymer phase. Such compatibilized composite films are brought by the GPMS that offers enough bonds/interactions between the organic and inorganic phases. These two factors would lead to the reduction of the average particle size of silica and good dispersion of such reduced silica particles in the polymer matrix. However, the overall phenomenon observed could be interpreted in terms of increasing domain size of silica that result in high scattering. Similar results were reported previously for the polyimide/silica-based composites [34,38,39]. 

### 3.4. FT-IR Spectra

FT-IR spectra of pure PI and PI-TSi-GPMS50 composites are shown in Figure 6. Some distinguish peaks near 1782 cm*^−^*^1^ (C=O asymmetrical), 1712 cm*^−^*^1^ (C=O symmetrical), 1367 cm*^−^*^1^ (C-N stretching), and 727 cm*^−^*^1^ (C=O bending) are the main characteristic adsorption peaks of polyimides [34,35,36,37,38,39,43,44]. In contrast, another characteristic peak at 1680 cm*^−^*^1^ for PAA is absent, which suggests that samples are fully imidized under employed conditions. Importantly, the peak in the range of 1000 to 1100 cm*^−^*^1^ has become very broad for PI-TSi-GPMS50 composite, which is due to the presence of the Si–O–Si stretching bonds, in good agreement with earlier studies on PI/silica type composites [34,35,36,37,38,39]. The presence of silica particles was also confirmed by SEM images in Figure 4. The obtained FT-IR results are well-consistent with previous studies on the formation of polyimide and composites [34,35,36,37,38,39,43,44].

### 3.5. Thermal Properties

Thermogravimetric profiles of pure PI, PI-TSi, and PI-TSi-GPMS-50 are displayed in Figure 7. The negligible weight loss below 100 °C suggests the absence of a noteworthy amount of water/moisture and solvents [38,39,40,41,42,43,44,45,46,47]. Figure 7 indicates that thermal stability of the pure PI is found to be enhanced by 24 °C when PI contains pure silica such as PI-TSi composite, due to the incorporation of silica and then decreased again for sample PI-TSi-GPMS50, associated with the presence of a large glycidoxypropyl segment in GPMS and the flexible alkyl segment started to degrade at 226 °C as shown in Figure 7. It should be noted that thermal degradation for flexible alkyl segments containing polymer-composites usually begins degrading after 200 °C, which also depends on the nature of the alkyl segments attached to the silica/organosilica framework. The T_d5_ temperatures of the pure PI and composites are reported in Table 1. The T_d5_ of the pure PI and PI-TSi-GPMS50 indicate that the PI-TSi-GPMS50 sample was lower than that of the pure PI, because of thermal degradation of the alkyl propyl segment of GPMS, which is found to be less thermally stable in Figure 7. These results are found to be consistent with previous results on PI/silica or PI/silsesquioxane or PI/POSS [18,19,20,21,22,34,35,36,37,38,39,40,41,42,43,44].

### 3.6. Dielectric Properties

The dielectric constants of the PI and its composite films largely depend on the experimental conditions including the type of dianhydride and diamine, size of the domain, morphology, final formed composite structure, and type of organosilica [34,38,39,45,46,47]. This study has used pure silica from TEOS and GPMS-modified silica to investigate how they affect the final value of DC of PI. Figure 8 shows the DC values of PI and its composite films. It is seen that the incorporation of silica into pure PI increases the DC, which is due to the inherent higher DC of silica 4 and large silica particles usually increase the free volume, which could also be occupied by small water molecules/moisture that would prelude an increase in the DC because of its high DC value [34,38,39,45,46,47]. On the other hand, the PI-TSi-GPMS25, which is prepared by the partial replacement of TEOS with GPMS, started to decrease the DC value by a small amount, and also further DC values were found to be significantly reduced for PI-TSi-GPMS50 and PI-TSi-GPMS75. Based on the previous reports [39,46,47], the observed DC trend in Figure 8 could probably be interpreted by considering the increased free volume, impact of organic propyl segment of GPMS, and final domain size of particles and composite phases. Tsai et al. have reported the DC values of various PI/phenyltrialkoxysilane (PTS) nanocomposites in which p-aminophenyltrimethoxysilane was used to facilitate bonding and interaction between the phenyltrialkoxysilane and ODPA-ODA phase [39]. Similarly, the introduction of trialkoxysilanes such as organosilica and silica-based bonded organic segments including phenyl, methyl, vinyl, octakis(glycidyldimethylsiloxy), functional allyl alcohol, octa(aminophenyl), into polyimide was found to decrease the final DC values of the composite films due to the presence of organic groups that show hydrophobicity. In addition, introducing more GPMS content means incorporating more hydrophobic propyl segments in the final composites. This modification contributes to reducing the DC of the composites and the final free volume will possibly be affected by the hydrophobicity of the propyl organic segment of GPMS like-domain. These results are well-consistent with previous reports on the trialkoxysilanes/POSS with various organic groups [34,38,39,45,46,47]. 

## 4. Conclusions

In this paper, we have demonstrated the PI-silica and GPMS-modified silica composites. Based on the results, the following factors are crucial for developing compatibilized composites:

(i) The substitution of TEOS with GPMS can reduce the formation of large silica particles by using a large number of hydroxyl groups and can also form H-bonds with anhydride, carbonyl, carboxyl, and terminal amine groups in the polyimides/polyamic acids.
(ii) The organic segment of GPMS can contribute significantly to bringing compatibilization between the two phases.
(iii) Epoxy group of GPMS will contribute to the formation of H-bonds or networks with carboxyl acid groups or terminal amine groups of the PAA for the compatibilized composites (homopolymerization and their impacts on the PI-silica composite cannot be ruled out for this kind of system).
(iv) SEM images suggested that the size of the silica particles in the PI/TSi composites was remarkably decreased by the addition of GPMS. Overall, it could be suggested that the incorporation of GPMS can produce compatibilized PI-silica composites with improved optical transmittance and controlled dielectric constants and all of the reported composites have shown *T*_d5_ more than 460 °C. The above investigations will help increase the feasibility of using PI-silica composites in many useful structural applications such as in high-temperature manufacturing plants, electronic packaging, aerospace, and automotive industries.

## Figures and Tables

**Table 1:** Physical, optical transmittance, and the relevant TGA temperature that triggered 5 wt% weight loss of the samples.

Sample Code	Physical Appearance	Optical Transmittance (T%)	5 wt% Weight Loss from TGA (°C) (*T*_d5_)
PI	Transparent and flexible	88	576
PI/TSi	Opaque and flexible but rigid	45	600
PI/TSi-GPMS25	Opaque and flexible	45–47	Not done
PI/TSi-GPMS50	Translucent	60	460
PI/TSi-GPMS75	Translucent	62	Not done

Note: Physical appearance: eye observation; T%: Optical transmittance at 638 nm. The numerical number at the end of the sample codes is the amount of TEOS that was replaced with GPMS. *T*_d5_: obtained from TGA analysis.

## Data Availability

The data presented in this study are available in this study. Additional information could be available on request from the corresponding author.
